# Analytical Modeling for a Video-Based Vehicle Speed Measurement Framework

**DOI:** 10.3390/s20010160

**Published:** 2019-12-26

**Authors:** Mattias Dahl, Saleh Javadi

**Affiliations:** 1 Department of Mathematics and Natural Sciences, Blekinge Institute of Technology (BTH), 37179 Karlskrona, Sweden; 2 Department of Mathematics and Natural Sciences, Blekinge Institute of Technology (BTH), 37435 Karlshamn, Sweden; saleh.javadi@bth.se

**Keywords:** vehicle speed measurement, temporal sampling, analytical modeling, motion analysis, pattern recognition, image processing

## Abstract

Traffic analyses, particularly speed measurements, are highly valuable in terms of road safety and traffic management. In this paper, an analytical model is presented to measure the speed of a moving vehicle using an off-the-shelf video camera. The method utilizes the temporal sampling rate of the camera and several intrusion lines in order to estimate the probability density function (PDF) of a vehicle’s speed. The proposed model provides not only an accurate estimate of the speed, but also the possibility of being able to study the performance boundaries with respect to the camera frame rate as well as the placement and number of intrusion lines in advance. This analytical model is verified by comparing its PDF outputs with the results obtained via a simulation of the corresponding movements. In addition, as a proof-of-concept, the proposed model is implemented for a video-based vehicle speed measurement system. The experimental results demonstrate the model’s capability in terms of taking accurate measurements of the speed via a consideration of the temporal sampling rate and lowering the deviation by utilizing more intrusion lines. The analytical model is highly versatile and can be used as the core of various video-based speed measurement systems in transportation and surveillance applications.

## 1. Introduction

Traffic surveillance systems collect and analyze road transportation data in order to improve road flow and safety. Since vehicles constitute the main component in road transportation, it is necessary to measure their respective parameters such as flow, speed, direction, and density. Early measurement methods were mostly focused on physical measurements, e.g., radar, laser, induction coil, or triple-loop sensors that, to some extent, required advanced equipment [1,2,3,4]. With advances in camera technology and image processing, it has been shown that these measurements can be taken effectively and efficiently using cameras as well [5,6].

The common configuration is to have a camera facing down at the road alongside a lane to capture video frames of vehicles. Then, consecutive frames are processed using computer vision techniques in order to calculate the vehicle’s speed. To do so, the vehicle’s location is extracted from the background in consecutive frames. Then, the vehicle’s actual displacement in the real world is found via the pixel displacement in the video frame [6,7,8,9]. In [6], a background model of the road is created and then the camera vibration is compensated to reduce the noise and consequently improve the measurement. In [7], a solar-powered automated speed violation detection system is presented. In this system, a binary difference image is created and then the location of the moving vehicle is extracted by finding the maximum and minimum variations in the binary difference image. In [8], a reference image is divided into three zones, and, after background subtraction, the coordinates of the centre of the vehicle are extrapolated to the reference image. A simulation model with two calibration lines and corresponding images is presented in [9]. In this simulation, the error rate is considered to be due to the imaging resolution, which is based on the distance between the camera and the vehicle. In all these methods, it is important to extract the pixels of the moving vehicle and avoid the detection of transient false objects such as shadows or noise [10].

There are also methods based on virtual line analyzers that are able to detect and track a vehicle as it crosses a virtual line and classify it based on color and size using background subtraction [11,12,13]. However, these methods have been introduced mainly to detect the presence of a vehicle on the road and not its velocity.

In [14,15], a three-frame difference method was introduced that computes the contour of a moving vehicle in three consecutive frames, whereas, in [15], the Horn–Schunck optical flow algorithm is used to calculate the displacement of a detected contour and, later on, to determine the speed of the moving vehicle. The moving object’s silhouette can also be considered to obtain a more precise speed measurement [16,17]. These methods extract discriminative features directly from all over a vehicle at different heights rather than from a specific horizontal plane.

Furthermore, there are approaches to tracking a specific region of a moving vehicle such as the license plate region [18,19]. Due to the perspective distortion, the projective transformation (inverse perspective mapping) can be computed using the camera’s parameters to obtain a more reliable conversion from the pixel displacement to the real-world displacement [18].

When considering these previous works, the need for an analytical model to serve as the core of these video-based speed measurement systems can be seen. Any such model should be able to address the main parameters contributing to the uncertainties in video-based measurements introduced as the result of the temporal sampling procedure and feature extraction. Since the captured video consists of consecutive frames in constant intervals, it is a discrete realization of a continuous phenomenon. Therefore, regardless of the implementation method, the analytical model should associate a probability density function (PDF) with the measured speed of a passing vehicle.

The analytical model proposed in this paper has the capacity to deal with the temporal sampling of the camera and the vehicle’s position extraction, thereby making it capable of measuring the speed in a controlled manner. This system utilizes several intrusion lines (two or more), along with their respective distances, in order to detect the movement pattern of a passing vehicle. Then, the proposed model evaluates the PDF of the possible speed using the detected input movement pattern.

This paper is organized as follows. In Section 2, the methodology of the proposed system is described in detail. It includes the analytical and simulation models, which uses MATLAB^®^ to measure the speed of the passing vehicle. The experimental results and discussion are provided in Section 3. Finally, the paper is concluded in Section 4.

## 2. Proposed Methodology

Video-based intrusion detection is a technique that is used to determine the entrance into, or crossing of, a vicinity on the part of an intruding object. There are many methods that can be utilized to realize this goal, and they are mainly employed by surveillance systems to detect any unauthorized entries into an area [20]. However, measuring the velocity of an intruding object is a complex task due to the camera’s temporal sampling and projection [17]. In the case of traffic monitoring, the complexity is increased due to the high speeds and frequency of the vehicles as well as the need for accurate measurements in a legal sense. Therefore, an analytical model is required to consider the uncertainties regarding video-based speed measurements, thereby making it possible to compute the precision and therefore improve the accuracy. In order to tackle the aforementioned issues, the following analytical model is proposed, and its reliability is demonstrated by comparing its results with the results obtained from the simulation model.

### 2.1. Analytical Model

**Figure 1:**
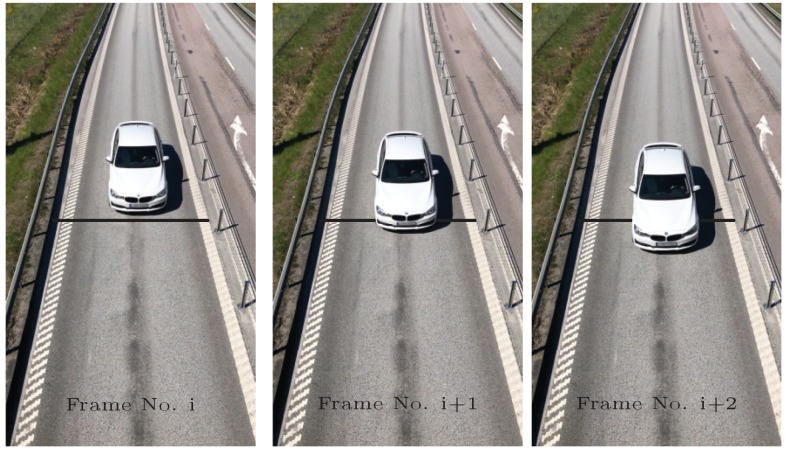
Discrete positions of a moving vehicle in consecutive video frames and its relative position with respect to an intrusion line.

**Figure 2:**
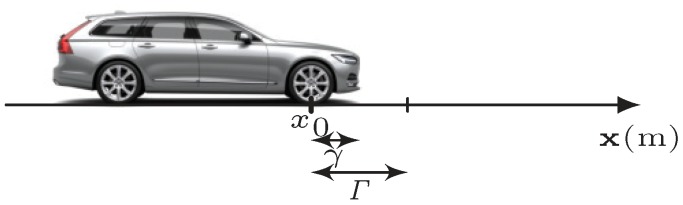
The detection distance Γ after the first intrusion line $x_{0}$ and the initial distance γ of the detected vehicle for a hypothetical speed *v*.

A vehicle appears in discrete positions in consecutive video frames due to the temporal sampling of the camera. Therefore, if the vehicle is detected crossing an intrusion line in a specific video frame, the vehicle’s position has a distance from the intrusion line at that moment; see Figure 1. This distance γ (m) is a random value within the maximum possible detection distance Γ (m), which is directly related to the hypothetical speed of the vehicle *v* (m/s) and the camera’s sampling time *T* (s),

(1) $$\begin{array}{c} \Gamma =Tv \end{array}$$

(see Figure 2). In the case where there are several intrusion lines, the vehicle should be detected independently after crossing each intrusion line. The travelling time of the vehicle between two intrusion lines can be obtained from the difference number of the frame indices and the camera’s sampling time.

**Figure 3:**
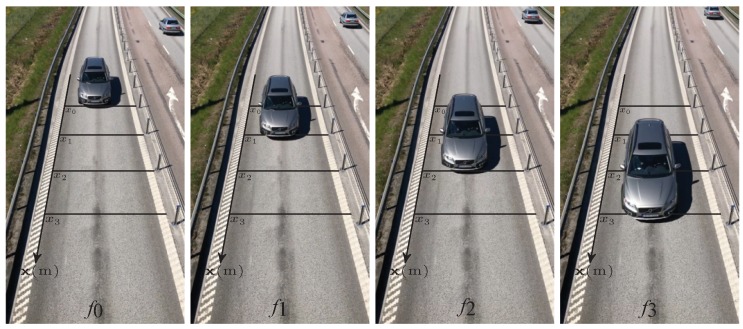
An example of a video-based speed measurement system with four intrusion lines placed at $x_{m}$ and the video frames where the intrusions are detected $f_{m}$.

Let us assume that the frame indices’ difference numbers $n_{m}$ between detections at intrusion lines are captured by the movement pattern vector $n=[n_{0},\;\ldots ,\;n_{M}]\in N$. In addition, the distances $d_{m}$ of the intrusion lines from the origin are contained in the intrusion lines distance vector $d=\left[d_{0},\;\ldots ,\;d_{M}\right]\in R_{0}^{+}$:

(2) $$\begin{array}{c} n_{m}=f_{m}-f_{0}, \end{array}$$

(3) $$\begin{array}{c} d_{m}=x_{m}-x_{0}, \end{array}$$

where m∈{0, 1, …, M}, M+1 is the number of intrusion lines, $f_{m}$ is the frame index number at which detection occurs, and $x_{m}$ (m) is the position of the intrusion line in the real-world coordinate system (see Figure 3). In the proposed model, the speed of the vehicle is assumed to be constant throughout the detection scene from $x_{0}$ to $x_{M}$.

The maximum number of intrusion lines is dependent on the minimum distance between any two consecutive intrusion lines. This minimum distance is constituted by the system to detect the highest possible speed. Thus,

(4) $$\begin{array}{c} d_{m}-d_{m-1}\geq Tv_{\max}, \end{array}$$

where m∈{1, …, M} and $v_{\max}$ is the maximum possible speed of any vehicle in the vicinity. This condition guarantees detection at all intrusion lines for the fastest vehicle with the maximum speed that the system is supposed to measure.

If $x_{0}$ is considered to be the origin ($x_{0}=0$), then the detection distance and the vehicle’s position at the detection moment at each intrusion line *m* in the real-world coordinate system at a hypothetical vehicle’s speed *v* can be defined as

(5) $$\begin{array}{c} h_{m}\left(x\right)=u\left(x-d_{m}\right)-u\left(x-d_{m}-Tv\right), \end{array}$$

(6) $$\begin{array}{c} w_{m}\left(x,\gamma \right)=\delta \left(x-\gamma -n_{m}Tv\right), \end{array}$$

where $h_{m}$ consists of two Heaviside step functions *u* and $w_{m}$ and a Dirac delta function δ.

Here, based on a detected movement pattern vector n for a given intrusion lines distance vector d, the aim is to find the probable speeds that satisfy this pattern of detected intrusions. Consequently, a PDF will be associated with the vehicle’s speed.

In order to detect the intrusion successfully, the vehicle’s position (i.e., $x=\gamma +n_{m}Tv$) should be within the detection distance for the hypothetical speed *v*. Therefore, based on the detected n, the intrusion conditions should be satisfied for each line simultaneously as follows:

(7) $$\begin{array}{ccc} g(v|n,d) & = & \int_{0}^{Tv}\left(\overset{M}{\underset{m=0}{\Pi }}h_{m}\left(x\right)w_{m}\left(x,\gamma \right)\right)\;d\gamma \\ & = & \int_{0}^{Tv}\left(\overset{M}{\underset{m=0}{\Pi }}h_{m}\left(x\right)\delta \left(x-\gamma -n_{m}Tv\right)\right)\;d\gamma . \end{array}$$

Since the Dirac delta function δ is equal to zero everywhere except at $x=\gamma +n_{m}Tv$, the above equation can be reduced to

(8) $$\begin{array}{c} g\left(v|n,d\right)=\int_{0}^{Tv}\left(\overset{M}{\underset{m=0}{\Pi }}h_{m}\left(\gamma +n_{m}Tv\right)\right)\;d\gamma . \end{array}$$

The initial detected distance of the vehicle γ cannot be out of the range of the detection distance (0, Tv); otherwise, no detection is possible. The PDF is given by

(9) $$\begin{array}{c} f_{V}\left(v|n,d\right)=\frac{g(v|n,d)}{\int_{v_{\mathrm{lower}}}^{v_{\mathrm{upper}}}g\left(v|n,d\right)\;dv}, \end{array}$$

where $v_{\mathrm{lower}}$ and $v_{\mathrm{upper}}$ are the lower and upper bounds of the hypothetical speeds with g(v|n, respectively;d) is greater than zero and $f_{V}\left(v|n,d\right)$ is the PDF for the stochastic speed *V*. Accordingly, the expected speed can be determined as

(10) $$\begin{array}{c} E\left[V\right]=\int_{v_{\mathrm{lower}}}^{v_{\mathrm{upper}}}vf_{V}\left(v|n,d\right)\;dv, \end{array}$$

where E[V] is the vehicle’s expected speed with the given movement pattern vector n and the intrusion lines distance vector d. Furthermore, the variance and the standard deviation σ are obtained as

(11) $$\begin{array}{c} \mathrm{Var}\left[V\right]=\sigma_{V}^{2}=\int_{v_{\mathrm{lower}}}^{v_{\mathrm{upper}}}{\left(v-E\left[V\right]\right)}^{2}f_{V}\left(v|n,d\right)\;dv. \end{array}$$

It should be noted that the proposed model, as a core measurement tool, can be used for multiple vehicles and multi-lane roads. In these cases, the PDFs of the vehicles’ speeds are still calculated based on their movement pattern vectors n. Therefore, in the case of multiple vehicles within the detection region, one solution would be for each lane to have a separate corresponding system. Another solution is to add a tracking component to track each vehicle separately through the intrusion lines in order to obtain their movement pattern vectors. Then, the analytical model would compute the PDF and expected speed for every single vehicle individually.

### 2.2. Simulation Model

In this section, a simulation model is established that generates the probability distribution for a movement pattern vector n. First, it is initialized with the camera sampling time *T* and the intrusion lines distance vector d. Then, the PDF of the vehicle’s speed associated with n is obtained using two methods: the analytical model and the simulation model. The results are then compared for the numerical verification of the analytical model.

According to the analytical model, given the movement pattern vector n and calculating the respective functions (5)–(9), the probability of any given hypothetical speed is obtained and therefore the PDF of the vehicle’s speed can be found.

On the other hand, the movement of the vehicle is simulated for various speeds using different initial distances γ within the speed range. The intervals for the given hypothetical speeds *v* and the initial distance γ are kept very small to keep the measurements reliable. Next, based on the simulated movement of the vehicle, a movement pattern vector n is obtained. This movement pattern is then compared with the one used for the analytical model to evaluate the validity of the hypothetical *v* and γ. After many iterations, the probability for various hypothetical speeds and consequently the PDF of the vehicle’s speed are determined. Finally, the PDFs obtained from the simulation model and the analytical model are compared with each other in order to verify the analytical model.

**Figure 4:**
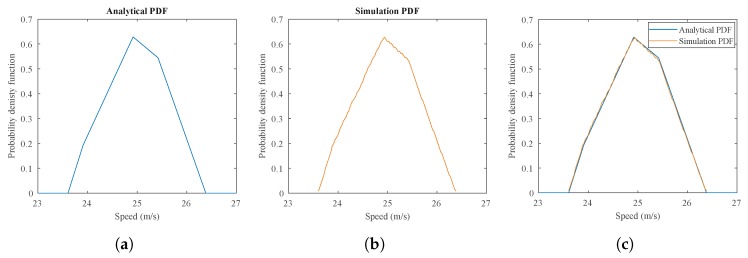
An example of the results obtained by the analytical and simulation models with n = [0, 6, 12, 18] and d= [0, 2.87 m, 5.95 m, 8.97 m] (**a**) the PDF from the analytical model; (**b**) the corresponding PDF from the simulation model; (**c**) the comparison between obtained PDFs.

The first simulation model is initialized with four intrusion lines d= [0, 2.87 m, 5.95 m, 8.97 m] and a camera sampling time T = 0.02 s. The speed range was selected from $v_{\min}\;=\;10$ m/s to $v_{\max}\;=\;40$ m/s within the interval Δv = 0.01 m/s. The initial distance γ is also considered to be within 0≤γ≤Tv with the interval of Δγ=0.001 m. Therefore, various movement pattern vectors are simulated; consequently, their associated PDFs are obtained. As an example, in Figure 4, the simulation result for a movement pattern vector n = [0, 6, 12, 18] is obtained and compared with the PDF from the analytical model for a video-based speed measurement system. As can be seen, the simulation’s PDF is indeed very similar to the analytical model’s PDF. With further increases in the number of speed steps, the PDF generated by the simulation model would become identical to the analytical model’s PDF. Therefore, the validity of the analytical model is supported by the simulation results.

**Figure 5:**
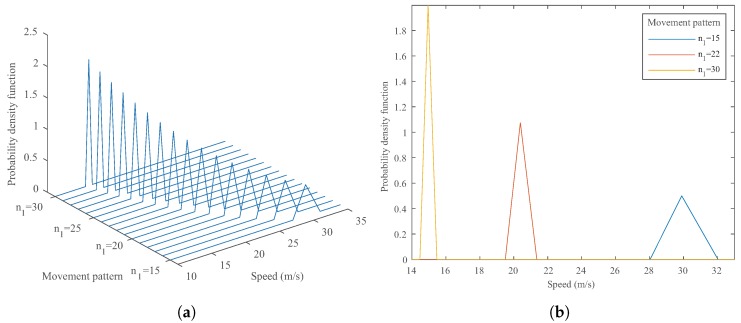
The performance evaluation graphs (**a**) For various input patterns for two intrusion lines. (**b**) Another illustration of three different movement patterns for two intrusion lines and their corresponding PDfs.

Furthermore, for a simple scenario involving only two intrusion lines d = [0, 9 m] and the camera sampling time T = 0.02 s, the PDFs corresponding to different movement patterns $n\;=\;[n_{0},\;n_{1}]$ generated by the analytical model are illustrated in Figure 5. It can be seen that slower vehicles have smaller variances and sharper PDF graphs than faster vehicles. As expected, this finding emphasizes that, in order to achieve higher accuracy, either the sampling time should be reduced or the distance between intrusion lines should be increased. However, more intrusion lines could also be utilized within the detection region to improve the accuracy, as shown in the next section.

## 3. Experimental Results and Discussion

As a proof-of-concept, an experiment was conducted on a major highway using a GPS-equipped vehicle monitored by a camera. A recording was made using an off-the-shelf camera with a frame rate of 50 fps, and the size of the input frame was 960×540 pixels. The vehicle passed through the detection region at known speeds measured by the mounted GPS, establishing the ground truth. The aim of this experiment was to verify the proposed model’s measurements of the vehicle’s speed and indicate the accuracy and deviation of the measurements based on the number of intrusion lines.

**Figure 6:**
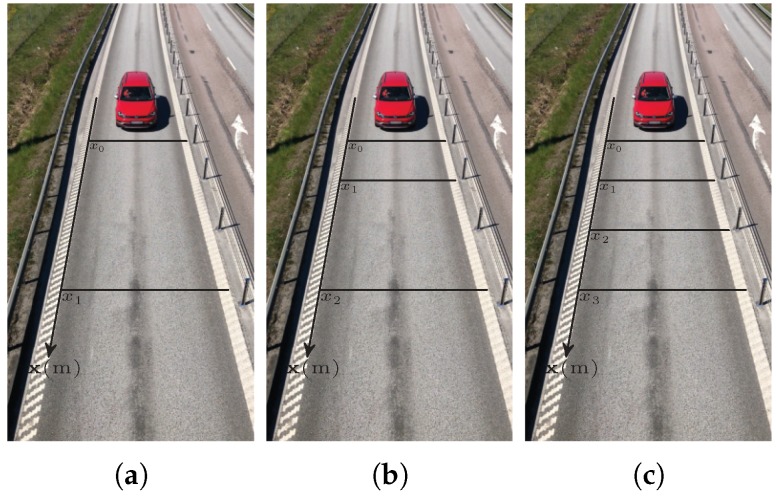
Intrusion lines configurations, (**a**) two intrusion lines with d = [0, 8.97 m]; (**b**) three intrusion lines with d = [0, 2.87 m, 8.97 m]; (**c**) four intrusion lines with d = [0, 2.87 m, 5.95 m, 8.97 m].

The detection region was constructed with three different intrusion line configurations for each attempt, as demonstrated in Figure 6. As the vehicle crossed the intrusion lines, a movement pattern vector n was created and fed into the analytical model. The analytical model then computed the PDF of the vehicle’s speed based on the movement pattern vector n, the intrusion lines distance vector d, and the camera sampling time *T*.

**Figure 7:**
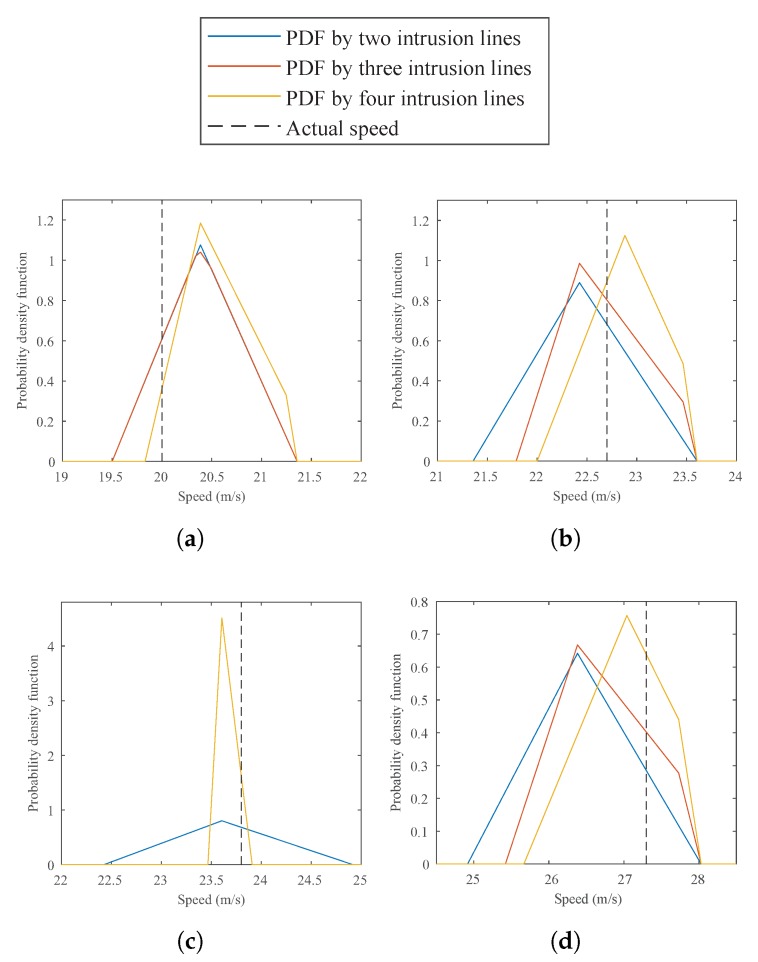
The computed PDFs for each attempt using different intrusion lines configurations (as described in Figure 6), (**a**) actual speed 20.0 m/s; (**b**) actual speed 22.7 m/s; (**c**) actual speed 23.8 m/s; (**d**) actual speed 27.3 m/s.

Table 1, Table 2 and Table 3 show the measured speed range (lower and upper bounds), the expected value (E[V]), the standard deviation σ, and the error rate (the absolute difference between the actual and expected speeds for each attempt). As can be seen, all the actual speeds are indeed within the estimated speed ranges, verifying the analytical model. Furthermore, using more intrusion lines yielded higher accuracy and less deviation for each measured speed in comparison with fewer intrusion lines, as shown in Figure 7. On the third attempt (see Figure 7c), the PDFs generated by using three and four intrusion lines are identical, and these PDFs provide better estimations than the PDF generated by two intrusion lines.

Table 4 presents the average error rate of the proposed method in comparison with those of previous works in [6,7,17]. As can be seen, our proposed system with both three and four intrusion lines has obtained better accuracy. However, these previous works did not provide the uncertainty in terms of the standard deviation of their measurements, with the exception of [18], in which the standard deviation is presented as 1.36 km/h. In this case, when our system used four intrusion lines, it performed better with a standard deviation of 1.26 km/h (0.35 m/s).

One of the key points of the proposed analytical model is that it has considered how the camera sampling rate and detection scene affect the final measurement accuracy. It has been shown that the camera sampling time *T* has a close relation with the intended speed range *v* to be detected. In the proposed analytical model, these two factors are combined as Γ = Tv, and both factors contribute greatly to the final measurement accuracy. Therefore, the first step in implementing any speed measurement system is to set a proper sampling rate or camera frame rate for the speed of interest. The second step is then to design a suitable intrusion line distance vector d to obtain higher accuracy and less deviation. As described earlier, the movement pattern vector n is based on the difference numbers of the frame indices, which are used for detecting intrusions in specific video frames. Having several intrusion lines means a longer movement pattern vector; consequently, more equations must be satisfied simultaneously in (8), which, in turn, leads to more accurate measurements and smaller PDF support.

It is also worthwhile to mention that the proposed analytical model is indeed compatible with existing methods and can improve the deviation and accuracy since all these methods eventually measure a displacement over a period of time based on the number of frame differences. Therefore, using the proposed model and several intrusion lines lead to a PDF of the speed rather than a single measurement of that speed.

## 4. Conclusions

In this paper, an analytical model is introduced for video-based speed measurement that is based on intrusion lines, handheld computers, and an integrated camera. The proposed model considers the temporal sampling of the camera, which affects the uncertainty of the measurements. The relation between sampling time (camera frame rate), the number of intrusion lines, and the distances between these intrusion lines are investigated thoroughly within a mathematical framework. The final output of the proposed model is a PDF of the vehicle’s speed as it crosses the intrusion lines. In addition, a simulation model is used to generate vehicles passing through at various speeds and different initial distances to obtain the PDF of the vehicle’s speed. The outputs of the analytical and simulation models were identical, verifying the analytical model and the applicability of the method itself. Furthermore, the experimental results for the analytical model demonstrated a promising performance, as all the actual speeds were within the obtained supports of the PDFs. Moreover, it was shown that the accuracy and the deviation of the measurements were improved by utilizing more intrusion lines within the detection region when using the analytical model. However, the proposed model offers a fair measurement of the speed with just two intrusion lines. The established framework is general and can easily be extended to several lines based on the discussed constraints and prior information about the vicinity.

## Figures and Tables

**Table 1:** The performance evaluation of the proposed method with two intrusion lines’ configuration.

Actual Speed (m/s)	Lower-Upper (m/s)	Expected Value (m/s)	Standard Deviation (m/s)	Error (%)
20.0	19.5–21.4	20.4	0.38	2.00
22.7	21.4–23.6	22.4	0.46	1.32
23.8	22.4–24.9	23.6	0.51	0.84
27.3	24.9–28.0	26.4	0.63	3.29
			av.: 0.50	av.: 1.92

**Table 2:** The performance evaluation of the proposed method with three intrusion lines’ configuration.

Actual Speed (m/s)	Lower-Upper (m/s)	Expected Value (m/s)	Standard Deviation (m/s)	Error (%)
20.0	19.5–21.4	20.4	0.38	2.00
22.7	21.8–23.6	22.6	0.40	0.44
23.8	23.5–23.9	23.7	0.09	0.42
27.3	25.4–28.0	26.7	0.58	2.20
			av.: 0.40	av.: 1.28

**Table 3:** The performance evaluation of the proposed method with four intrusion lines configuration.

Actual Speed (m/s)	Lower-Upper (m/s)	Expected Value (m/s)	Standard Deviation (m/s)	Error (%)
20.0	19.8–21.3	20.5	0.34	2.50
22.7	22.0–23.6	22.9	0.35	0.88
23.8	23.5–23.9	23.7	0.09	0.42
27.3	25.7–28.0	27.0	0.51	1.10
			av.: 0.35	av.: 1.17

**Table 4:** The performance comparison of the proposed method with previous works.

	Method [6]	Method [7]	Method [17]	Our Method
**Error rate (%)**	4.35	1.97	1.82	1.17
